# Beef Breeding Systems and Preferences for Breeding Objective Traits

**DOI:** 10.3390/ani15152175

**Published:** 2025-07-23

**Authors:** Zuzana Krupová, Emil Krupa, Michaela Brzáková, Zdeňka Veselá, Kamil Malát

**Affiliations:** 1 Institute of Animal Science, Přátelství 815, Uhříněves, 104 00 Prague, Czech Republic; krupa.emil@vuzv.cz (E.K.); mbrzakova781@gmail.com (M.B.); vesela.zdenka@vuzv.cz (Z.V.); 2 Czech Beef Cattle Association, Těšnov 17, 110 00 Prague, Czech Republic; info@cschms.cz

**Keywords:** production systems, management, marketing, breeding objective traits, cluster analysis

## Abstract

Our study aimed to identify the overall and cluster-specific characteristics of Czech beef cattle breeding systems. We used data from an online survey to ascertain farmers’ preferences in breeding objectives. Considering various evaluation criteria and clustering approaches in 41 farms, three beef systems were defined according to herd size, management, marketing, breeding strategies and structures, and farmer age. Breeding values and performance were jointly used as the primary information in all three systems. Cow temperament and calf viability, maternal fertility and longevity, and animal health were found to be the most important traits. Cluster 1 represents pure-breeding farms that specialize in producing breeding animals. Farms in clusters 2 and 3 combined pure- and crossbreeding strategies with production, which was partially (cluster 2) and fully (cluster 3) diversified for all beef categories. Farms also prioritized calving performance and calf growth (clusters 1 and 2) and exterior traits (cluster 3). Production type scores significantly (*p* < 0.05) differed in clusters 3 (4.12) and 2 (3.25). The proportion of production, functional, and exterior trait categories was 12:37:51, with low variability among clusters (±1 to 2 percentage points). The inter-cluster comparison showed that specific characteristics were compatible with certain breeding goal trait preferences.

## Simple Summary

Breeding system characteristics and breeding objective traits are key elements of livestock breeding strategies. We carried out an anonymous online survey to describe these features in Czech beef farms. The surveyed farms were shown to be representative of local production conditions. Regarding breeding objectives, the top traits that are currently included in genetic evaluation were confirmed, and some novel traits were identified. Clustering approaches allowed us to understand the beef farms and their breeding objectives more precisely from a breeding and selection point of view. Three beef systems—specific in terms of both farm characteristics and preferred traits—are considered for further breeding strategy adjustment. These findings enable targeted breeding programs, enhancing genetic evaluation and sustainable beef production in the Czech Republic.

## 1. Introduction

Cattle production systems generally differ regarding production conditions, breed structures, market orientation, decision process-determining parameters, and many other features. Cluster analysis, which originated from application areas such as biology and is regarded as a very useful data-mining tool, allows for the assessment of many aspects of cattle farming [1]. Generally speaking, it sorts objects into groups (clusters) based on their similarity [1].

This instrument has been widely implemented in recent studies evaluating cattle farming, mainly for herd classification and animal breeding and selection. Cluster analysis was carried out to establish the typology and basic characteristics of suckler farm systems, evaluating their viability and sustainability by considering various production and economic determinants [2]. Similarly, clustering was applied to provide a novel classification of small farms in Europe, taking into account their typology and regional-level role for further policy intervention [3]. A phenotype evaluation of both pure- and crossbred Charolais cows was provided to observe variance within varieties, find phenotypically different types within these groups, and create an appropriate ranking for selection purposes [4]. Multi-algorithm automatic milking system data clustering aimed to assess cow productivity by group, as well as their stability over time for selection purposes [5]. Clustering was applied in the genetic evaluation of beef cattle herd weaning weights in order to consider genotype–environment interactions and identify variables more precisely describing individual herd conditions [6]. Similarly, clustering strategies were implemented to improve genetic evaluation accuracy based on the social–ecological and herd characteristics of small dairy cattle farms in alternative husbandry systems [7]. Farms using automatic milking systems were clustered into groups with unique management styles, challenges, and production characteristics [8].

Beef cattle systems in the Czech Republic cover more than 220 thousand suckler cows of 25 breeds located across more than 8 thousand farm units [9] with variable conditions; half of these are natural constraint areas (formerly less favored areas [10]). Farms may be located at altitudes from 300 to 700 m (74%) or over 700 m (21%) above sea level [11]; up to 50 cows is the most common number of animals (88%) [9]. About 11% of all suckler cows are included in routine beef performance records [9], which are key in practical breeding and selection. Local beef cattle populations can be enhanced through breeding programs in which values for over twenty traits are routinely estimated [12]. Some simple selection indices have been suggested for examining calving performance and calf growth [13]. Nevertheless, in recent times, the breeding program continues to be enriched through the incorporation of new functional traits into breeding value (BV) estimation [14], the application of genomic data [15,16], and the calculation of economic values [17]. To more comprehensively define breeding strategies and construct selection indices in a more practically applicable way, further details of breeding system characteristics are needed. They should reflect, i.e., the management, marketing, and breeding strategies; breed structure; and herd size. At the same time, farmers’ preferences for breeding objective traits should be respected. Both parameters are currently unknown and need to be defined for the local population. Moreover, they are necessary for appropriately setting input parameters to calculate the trait economic values using the bio-economic approach, as well as for the construction of customized breeding programs.

In this sense, we hypothesized that the survey brings recognition of the overall breeding system characteristics and farmers’ preferences in breeding objective traits, and secondly, the clustering approach enables us to define their specific patterns. Therefore, the main aim of this study was to define the overall and cluster-specific characteristics of breeding systems and evaluate preferences in breeding objective traits from the online survey to inform tailored breeding programs for Czech beef cattle conditions in the future.

## 2. Materials and Methods

In this study, we applied cluster analyses to allocate beef cattle farms into basic breeding systems, the overall and cluster-specific characteristics of which were defined. Secondly, the overall and cluster-specific breeding objective trait preferences were derived. Data were collected anonymously through the online questionnaire, which was made freely accessible to farmers via a link on beef breeder web pages (Czech Beef Cattle Association, CBCA) in 2024. Farmers were invited to follow the link to a short overview presenting the main aim of the survey. The final dataset comprised the herd characteristics and trait preferences (importance) of 41 Czech beef farms. Answer choices were predefined to minimize mistakes in filling out the questionnaire and to facilitate further data processing. There were a few open-ended questions so that breeders’ opinions can be considered outside of the predefined choices (i.e., when indicating animal breeding and selection decisions and when adding optional traits and their preferences to the questionnaire; details are given in the text below). The survey’s response structure (ratio) was subsequently analyzed to obtain a general overview of the beef farm structure, overall breeding and selection strategies, and farmers’ herd trait preferences. The overall sample size was built on the willingness of farmers to participate in the online survey, which could bring some limitation in the dataset’s statistical power. Nevertheless, any information concerning breeding system characteristics and farmers’ preferences for breeding objective traits is beneficial in covering the current absence of sufficient information needed for tailored breeding program settings.

### 2.1. Herd Characteristics

Basic herd characteristics involved management (e.g., conventional, ecological), marketing (e.g., selling of breeding animals, weaned calves, fatteners), breeding strategy (pure- and crossbreeding, or both), breeds farmed, herd size (number of cows), social aspects (age group of a farmer) and general decision-making when selecting animals (i.e., based on performance, breeding values (BVs), or both). The detailed structure of all herd characteristics and their options is presented in Table 1. This structure was primarily designed with animal breeding and selection in mind and in accordance with the most important determinants used in the herd clustering reviewed in [7]. Herd breeding strategies were characterized according to two variables: breeds used in pure- and crossbreeding (number in total) and the farmer-defined “first” breed (i.e., considered dominant for the given herd). Regarding animal breeding and selection decisions, farmers could add their own answers. Some farmers (19%) provided a specific list of performance traits, mentioning “breeding value” as a keyword. Mandatory (default) options given in the survey were clearly indicated; therefore, these answers could be cleanly assigned as decisions based on “achieved performance”, “breeding values (BVs)”, or their combination (“both BVs and performance”). Therefore, no answer was assigned the option “other” throughout the processing of survey data.

### 2.2. Herd Clustering Approach

In total, three clustering approaches—two centroid-based (k-means and partition around medoids (PAM)) and one hierarchical (represented by the agglomerative clustering (AHC))—were considered and tested in our study. K-means, PAM, and AHC belong to the most common clustering approaches applied in the literature mentioned above (e.g., [4,5,7]). In these studies, a detailed description of clustering principles was presented; a general description (mainly based on [1]) in this regard is provided in the following text.

K-means and PAM are centroid-based approaches, which divide *n* objects into *k* groups (partitions), where each partition represents a cluster and *k* ≤ *n*. The user should specify the number of clusters *k* as a termination condition. The initial representative objects (means and medoids) are iteratively replaced by other objects until the clustering quality is not improved (measured by the average dissimilarity between an object and the initial object of its cluster). PAM is more robust than k-means because outliers or extreme values influence a medoid less significantly than a mean. Approaches are generally categorized as fast, simple, and working well when finding clusters in small to medium-sized databases.

Agglomerative hierarchical clustering (AHC) combines data into a hierarchy (tree), which is visualized as a dendrogram using the bottom-up strategy. Each object considered a cluster is iteratively merged (nested) into larger clusters until all of the objects belong to a single cluster. The iterative merging process finds two close clusters to form one. The similarity between two clusters is measured by that of the closest pair of data points belonging to different clusters.

R Statistical Software version 4.4.3 (R Foundation for Statistical Computing, Vienna, Austria) [18] was used for all statistical analyses. The “cluster” R package (Version 2.1.8.1) [19] was used for processing input data through clustering analyses in all three evaluated methods (k-means, PAM, and AHC). The “factoextra” R package version 1.0.7. Ref. [20] was used to extract and visualize data clustering analysis results. The average silhouette width (ASW [21]), total within sum of squares (WSS), and gap statistics (GAP_k_ [22]) were used as criteria for evaluating the most appropriate clustering approach and defining the optimal number of clusters. Generally, the separation distance among clusters (ASW), their compactness, i.e., the closeness of data within clusters (WSS), and their optimal number were specified here using the “firstmax” approach (gap statistics). Using the WSS, the optimal number of clusters was also determined via the elbow method. In this way, we identified the number of clusters where the value of WSS started to decrease rapidly before reaching a plateau. According to [3], Pearson correlation coefficients, with *p*-values among the herd determinants, were calculated using the “rcorr” function in the “Hmisc” R package Version 5.2–3 [23] prior to clustering (Table S1). A ratio ranging from −0.272 to +0.507 indicated no strong correlation, and all variables were therefore used in ongoing analyses.

### 2.3. Farmers’ Preferences

Farmers’ preferences were collected in the second part of the survey. In total, 12 mandatory (default) trait groups and optional traits (added by farmers) were assigned scores from 1 (not important) to 5 (very important). Scores of 2, 3, and 4 represented “a little”, “medium (50:50)”, and “more important”, respectively. Traits related to calving performance, growth, body frame and capacity, muscularity, production type, maternal fertility and longevity, udder scoring and milkiness, temperament, bull fertility, and meat quality were considered mandatory. A structured list of mandatory and optional traits is presented in Table 2.

Mandatory group traits were defined according to those currently included in routine genetic evaluation and breeding value estimation for Czech beef cattle populations. Therefore, further detail on trait descriptions and evaluation methodologies can be seen in former studies (e.g., [14,15,24,25,26]) and on the CBCA web page (in Czech; https://www.cschms.cz/index.php?page=sle_info (accessed on 4 March 2025)). Likewise, trait definition and structure corresponded with the global standard for livestock data published in the International Committee for Animal Recording (ICAR) Guidelines [27]. In total, 19 farmers added optional traits without limiting their number per farm in the survey. During data checking, some responses were omitted from further processing: those inserted without a preference value (four answers) and infrequent traits (i.e., gestation length, feed conversion, and body condition added by four farmers in total). The most frequent traits, i.e., polledness and animal health (inserted by ten and eight farmers, respectively), were considered in further evaluation. The breeding goal traits were organized into three main categories—production, functional, and exterior. The assignment of individual traits (groups) into categories is shown in Table 2. Health was understood as a “functional” category, commonly assessing various traits such as maternal fertility with longevity and cow temperament with calf viability. Finally, relative category emphases per cluster were expressed as a percentage of all trait group scores of a given herd cluster. Correlation coefficients (r) and *p*-values among the farmers’ preferences and with the herd characteristics were calculated (presented in Tables S1 and S2), and analyses of the trait score differences between clusters in terms of effect sizes were provided.

## 3. Results

### 3.1. Overall Farm Characteristics and Trait Preferences

The overall farm characteristics are presented in Table 1, including management, marketing and breeding strategies, breed structure, herd size, farmer age group, and general decision-making when selecting animals. Farmers practiced both conventional and ecological management (44% and 49%, respectively, with 7% being in transition and mixed systems); they mainly produced (sold) breeding animals (34%) or diversified production (29%), weaned calves and/or fatteners, and combined pure- and crossbreeding in one herd (56% of farms). Almost 80% of surveyed farms had only one breed, with the most frequent being Aberdeen Angus (25%), Limousine (25%), Charolais (17%), and Beef Simmental (13%). The most common herd size was up to 29 cows (22% of farms), and 3/4 of farms had farmers aged up to 49 years. In terms of animal breeding and selection, farmers commonly made decisions based on both BVs and animal performance (73%). In terms of significant relationships among the herds’ characteristics (presented in Table S1), farms with a higher number of breeds (used both the pure- and crossbreeding strategy) were mostly running under the ecological management system, and farms with higher herd size produced (sold) weaned calves and/or fatteners in higher intensity.

**Figure 1:**
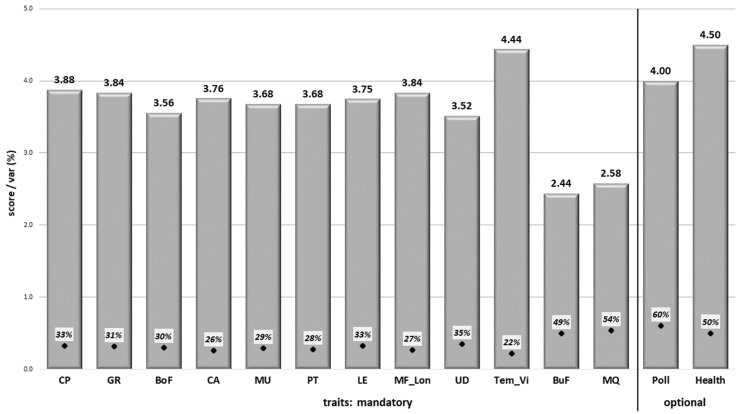
Mandatory and optional trait preference scores ^1^. ^1^ Score represents the importance of the trait group in the range: 1—not important, 2—a little, 3—medium (50:50), 4—more important, 5—very important. Var—variation coefficient (%) of the score assigned to traits. CP—calving performance, GR—growth, BoF—body frame, CA—capacity, MU—muscularity, PT—production (breeding) type, LE—legs, MF_Lon—maternal fertility and longevity, UD—udder scoring, TEM_Vi—cow temperament and calf viability, BuF—bull fertility, MQ—meat quality, Poll—polledness, and Health—animal health (for more details, see Table 2).

Figure 1 shows overall preferences (score) and their variability, as assigned by farmers to twelve mandatory groups and two optional breeding goal traits. Regarding the mandatory traits, results show that breeders give the highest importance to cow temperament and calf viability (score of 4.44), calving performance (3.88), and maternal fertility and longevity, along with growth (both 3.84) and body capacity (3.76). On the contrary, meat quality (2.58) and bull fertility (2.44) had lower importance, with the highest variability (52% on average) for the assigned score. For important traits, we saw higher agreement among preferences (variability of 28%). In terms of optionally added traits, polledness (4.00) and health (4.50) were appreciated by 44% of farmers, albeit with a relatively higher variability (55% on average). Significant correlation of assigned scores was mostly found among the exterior traits (body frame, capacity, production type, and legs; see Table S2). Simultaneously, body frame and capacity were more appreciated by farmers with higher herd size, and the trait production type less by those who used a pure-breeding strategy with a higher number of breeds. Correlations among the score assigned to evaluated traits and the farmer’s age indicated a negligible relationship (ranking from −0.18 to 0.30; all presented in Table S1).

### 3.2. Herd Clustering

**Figure 2:**
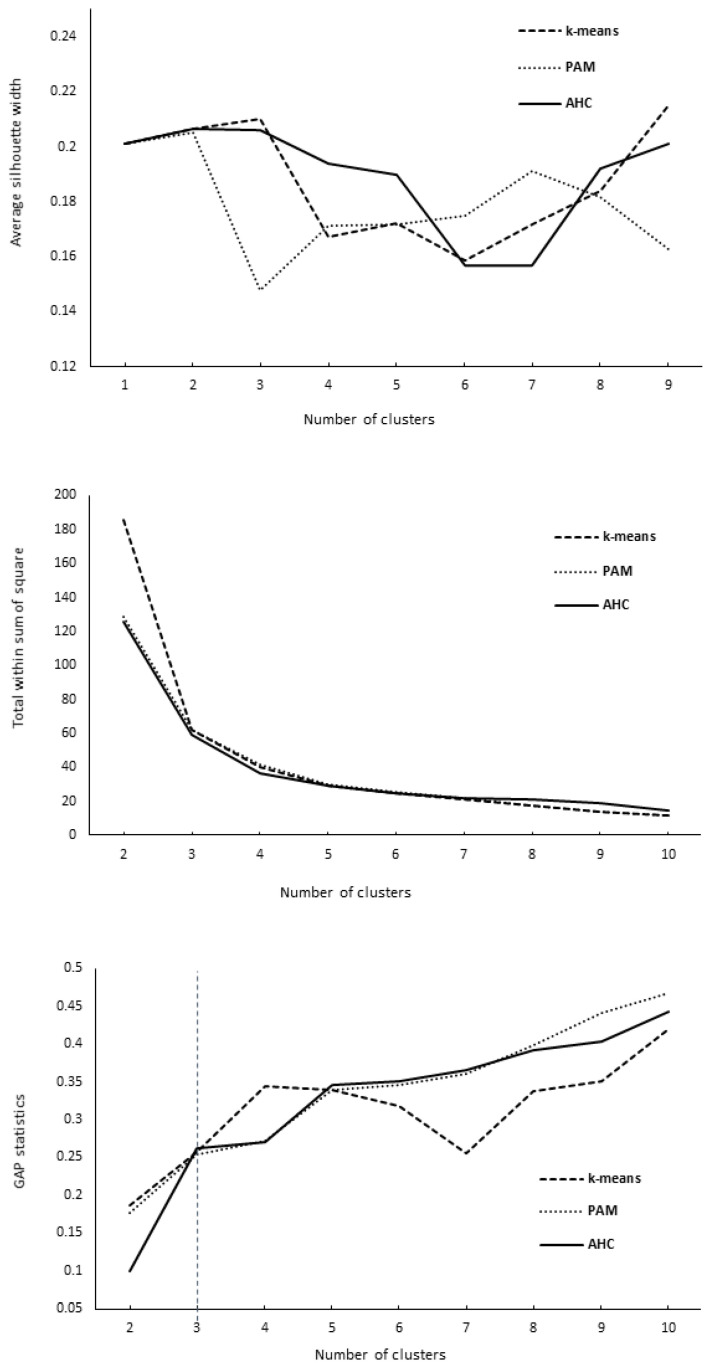
Average silhouette width, total within sum of squares, and gap statistics for the evaluated clustering methods ^1^. ^1^ K-means, PAM—partition around medoids, AHC—agglomerative hierarchical clustering. For a detailed description of methods, see the Materials and Methods section.

**Figure 3:**
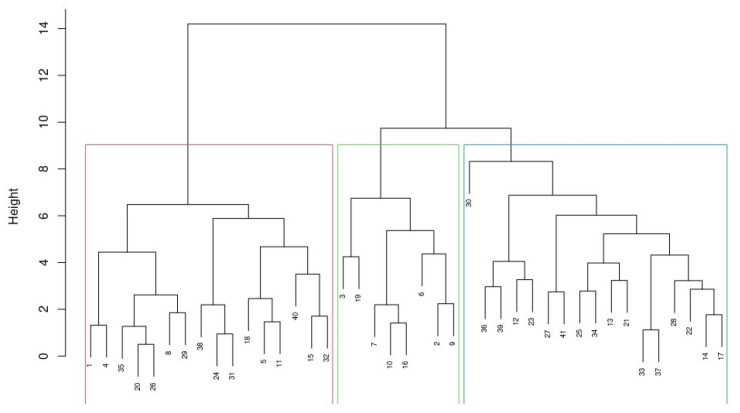
Allocation of herds into three clusters ^1^. ^1^ Blocks represent 16 (red), 8 (green), and 17 (blue) herds allocated to clusters 1, 2, and 3, respectively. In total, 41 herds were clustered. The individual herd numbers show the order in which the farmers participated in the survey.

**Figure 4:**
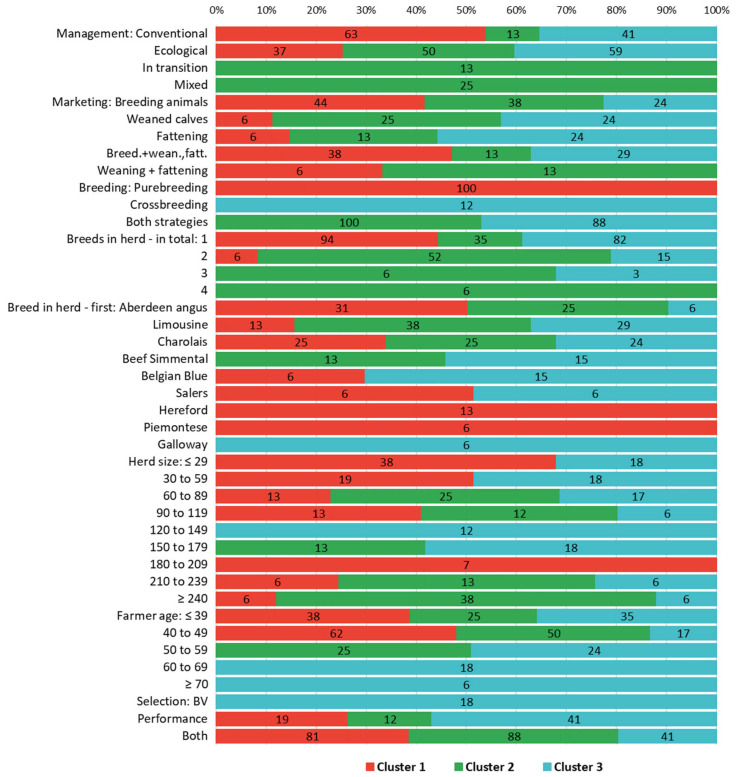
Beef cattle farm characteristics ^1^ (%) in clusters 1, 2, and 3. ^1^ More details on farms assigned to clusters and their characteristics can be seen in the Results section and in Figure 3, respectively. The ratio of respective characteristics sums 100% per given cluster and characteristic.

The average silhouette width, total within sum of squares, and gap statistics for the evaluated clustering methods are presented in Figure 2. The average silhouette width ranged from 0.149 (PAM with four clusters) to 0.215 (k-means with 10 clusters), indicating relatively close variability and similarity in terms of separation efficiency among evaluated clustering approaches. Regarding the total within sum of squares, compactness values among the cluster methodologies were also similar (see Figure 2). Such results were supported by gap statistics applying the “firstmax” or the first peak point method. Using the PAM and AHC approaches, the optimal number of clusters would be three. This is in accordance with the ASW plot, presenting the maximum separation distance in three clusters, and the WSS plots, where adding a cluster over three (“elbow” point) did not considerably improve compactness (see Figure 2). Overall, the AHC method showed a slight superiority for fitting our data, as suggested by the above-mentioned evaluation criteria. Herd clustering of the three groups using the AHC method is presented in the dendrogram (Figure 3) and cluster characteristics in Figure 4. In total, 16, 8, and 17 herds were allocated to clusters 1, 2, and 3, respectively.

Herds included in cluster 1 could be characterized as smaller farms (nearly 60% of them having up to 59 cows) that purely practiced organic or conventional management (conventional prevailing), principally applied pure-breeding (100%), and mostly farmed one breed (94% of all farms). Nevertheless, in total, seven different breeds were covered in this cluster. Farmers were in the younger age category (all up to 49 years) and mainly made selection decisions according to both BVs and animal performance (81%).

Cluster 2 included the largest farms surveyed (1/2 had over 210 cows), which were primarily ecological and mixed, combined both pure- and crossbreeding (100%), had only the most frequent breeds (Aberdeen Angus, Limousine, Charolais, and Beef Simmental), and two breeds per herd on average. Middle-aged farmers (75% were over 39 and up to 59 years old) managed farms and selected animals similarly to those in cluster 1, i.e., after considering the estimated trait BVs and animal performance (88%).

Herds in cluster 3 mostly covered medium-sized farms (53% ranging between 60 and 209 cows) that predominantly operated under ecological management (59%) and mostly combined pure- and crossbreeding (88%). Regarding breed structure, there was a relatively broad spectrum of breeds (seven in total), but typically only one breed in the herd (82%). Farmers belonged to all age categories, though nearly half of them were over 50 years of age and equally preferred using performance vs. a combination of BVs and performance when selecting animals (41% both).

### 3.3. Herd Cluster Preferences

**Figure 5:**
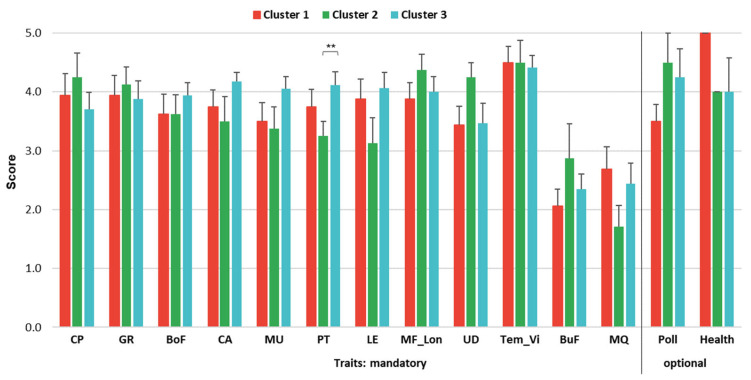
Trait preferences (score) ^1^ and respective errors in clusters 1, 2, and 3. ** *p* < 0.05. ^1^ Cluster-specific score assigned for traits: CP—calving performance, GR—growth, BoF—body frame, CA—capacity, MU—muscularity, PT—production (breeding) type, LE—legs, MF_Lon—maternal fertility and longevity, UD—udder scoring, TEM_Vi—cow temperament and calf viability, BuF—bull fertility, MQ—meat quality, Poll—polledness, and Health—animal health. For more detailed trait descriptions and cluster characteristics, please see Table 2 and Figure 4, respectively.

In terms of the cluster-specific preferences presented in Figure 5, production (breeding) type significantly (*p* < 0.05) differed in clusters 2 (score 3.25) and 3 (score 4.12). Similar to overall farmer preferences, cow temperament and calf viability received the highest mandatory trait scores in all three clusters (ranging from 4.41 in cluster 3 to 4.50 in the other two). Other top breeding goal traits also varied among clusters, such as calving performance and growth (3.94 both) in cluster 1, maternal fertility and longevity (4.38) and calving performance (4.25) in cluster 2, and body capacity (4.18) and production type (4.12) in cluster 3. Across all clusters, traits with the lowest preference scores were meat quality and bull fertility (ranging from 1.71 for the former to 2.88 for the latter, both in cluster 2). Regarding the optional traits suggested by the farmers, the assigned scores were relatively high, ranging from 3.50 (polledness) to 5.00 (health), both of which were reached in cluster 1.

A detailed description of the score differences between clusters (pairwise analyses) in terms of effect sizes measured by Cohen’s d confidence intervals for all traits is presented in Table S3. The results supported the significance differences of scores assigned to productive type trait, indicating a large effect (1.001), i.e., a substantial score difference between clusters 2 and 3. Additionally, a large effect was calculated for preference score in legs (0.849), meat quality (1.578), and polledness (0.949), all in cluster 2 vs. 3. Substantial differences in scores were also calculated for polledness (1.632) and health (0.929) between clusters 1 and 2, and for score of meat quality (1.438) in cluster 1 vs. 3.

The relative emphases on production, functional, and exterior trait categories within clusters 1, 2, and 3 were 13:38:49, 11:39:50, and 12:35:53, respectively. For the whole dataset, the main categories’ ratio was 12:37:51. Generally, the importance ratio was highest for the exterior (51% on average) and minor for the production (12% on average) trait categories, with relatively low variability among clusters (±1 to 2 percentage points in comparison to the average category ratio).

## 4. Discussion

Our study connects the overall and cluster-specific parameters of Czech beef cattle farms gathered through the online questionnaire. Valuable patterns could be identified from such a dataset source and size. It covers the evaluation and determination of the basic parameters of beef breeding systems, as well as farmers’ preferences in terms of breeding objectives. In the following, we will discuss these aspects in view of their practical application in animal breeding.

Overall farm characteristics

The ratio of conventional to ecological management (44:49, i.e., without mixed farms and in transition), as practiced in the surveyed beef cattle farms, was close to the 45:55 previously published regarding beef meat production structure [28]. Since 1990, ecological farming has generally expanded across Europe, aligning with the official strategy of “Farm to Fork”, whereby the share of ecological farms is expected to reach 25% by 2030 [29]. In terms of agricultural land area, ecological farming makes up 16% of the Czech Republic [30]. Nevertheless, beef farms are primarily located in more elevated areas [11] and border regions in the upper proportion of permanent grasslands [30]. The ratio of ecological areas is considerably higher in these regions (mainly ranking from 30% to 80%), with cattle as the most frequent livestock species [30].

Regarding marketing strategies, a third of farms’ incomes came from breeding animals, which corresponded well with the main objective of our survey focused on animal breeding and selection. Secondly, farms have diversified sources of revenue: breeding animals (bulls for AI/natural mating, heifers), weaned calves, and/or fatteners (29%). Diversified production could bring many benefits to beef farmers, reducing the risks associated with the farm business in terms of stable income [31], overall output value, investment return, and farm sustainability [32]. In accordance with the aforementioned, the breeding strategies of the surveyed farms mostly combined both pure- and crossbreeding in one herd (56%). Generally, a higher supply variability on the market (breeding and carcass animals), crossbred meat quality and yield, and the possibility of practical production applications could play important roles in these strategies.

The breed structures reported in our survey corresponded to the official cow numbers recorded by [9], in that Limousine, Charolais, Aberdeen Angus, and Beef Simmental were the most common. In terms of cows included in the 2024 performance testing, the list of the most popular breeds was similar; numbers of Charolais, Aberdeen Angus, Beef Simmental, and Limousine (based on own calculations from the database provided by the CBCA) have remained stable over the last decades [10]. The proportion of surveyed herds predominantly farming one breed (80%) was close to the 86% calculated from the CBCA database (relevant for herds with the most frequent breeds, according to our own calculations).

The herd size structure of the survey participants was relatively balanced (37% had up to 60 cows, 44% up to 210 cows, and 19% had larger farms, as shown in Table 1). This is in accordance with the authors of [33], who reported corresponding beef herd size ratios of 25%, 35%, and 40%, respectively. This balanced herd structure also aligned with the authors of [3], who stated that although farm sizes have decreased in recent decades, large-scale farms still prevail in Czech agriculture. Nevertheless, according to the central animal recording databases [9], small-scale farms dominated (53% up to 10 cows) and only 10% of farms had over 200 cows in 2024. This difference could possibly be due to the different data sources; the central database covered all farms, i.e., those owning one-cow herds as a hobby or as a secondary income activity. Such farms would probably have a minor emphasis on animal breeding and selection, although they would still receive practical benefits from enhanced breeding programs.

The most common farmer age calculated using our survey (75% up to 49 years) corresponded to the overall agriculture employee structure published by [30], where the majority (i.e., 58%) was up to 49 years old. However, the proportion of the following age category (over 50 years) is obviously lower in our survey (22%) than in the cited study (nearly 42%). The possible reason for this is the natural generational change in beef cattle farmers, which may have taken place with a slightly higher intensity (especially in terms of the position of farm owner/manager) compared to all other agriculture workers. In this context, the authors of [30] noted that generational change has been significantly disrupted in the last decades; the proportion of workers in older age categories has gradually increased, and this problem is common across agriculture in most EU countries. The online form of the survey, perhaps making it more accessible and easy to use for younger breeders, could have partially impacted the age structure found.

Farmers who participated in the survey mostly selected animals according to both BVs and performance (73%). This result seems to be positive from a breeding and practical point of view, showing that farmers have confidence in BVs, complementing it with or assessing it against measured performance. This attitude helps in building a valuable foundation for further breeding program enhancements.

Overall trait preferences

The identified overall farmer preferences correspond to the top traits considered in the genetic evaluation of local beef cattle populations [12]. The scores assigned to calving performance and growth positioned them amongst the four most important traits (Figure 1), followed by exterior or linear scoring traits (such as body capacity and productive type), which have long occupied a stable place in genetic evaluation across the globe [6,24,34]. Further traits that were highly preferred by farmers were cow temperament and calf viability, calving performance, and maternal fertility and longevity. These trait groups have recently been incorporated into local performance testing and evaluation [15,26] and feature in the evaluation of other beef populations [35,36]. As such, they would probably play an important role in further breeding program enhancements.

The same is true of the relatively high-scoring metrics of polledness and animal health, as directly suggested by farmers (Figure 1). Generally, polled cattle breeding brings farmers many benefits in terms of management, injury prevention, overall animal welfare, and public attitude. Polledness also has measurable economic benefits in terms of reducing dehorning costs (labor, material, veterinary treatment) and increasing the market value of genetically polled animals [37]. This trait is therefore a suitable candidate for animal breeding and selection. In this context, some specifics of the genetically polled Aberdeen Angus breed should be mentioned. Therefore, some farmers (mainly of Limousine, Beef Simmental, and Charolais) added this breeding objective when filling out the survey. In current breeding practices, calf survival is evaluated for animal health in local beef populations, with limb assessment to be used as an indicator of beef cattle resistance. Moreover, some specific diseases such as Charolais ataxia (mentioned by one of the survey respondents), diarrhea (causing calf loss), and cow respiratory diseases [11] could be considered here. In terms of the optional traits, gestation length, feed conversion, and body condition were also mentioned by farmers. Based on very low frequency (added by four farmers in total), they were omitted from further evaluation. Nevertheless, they should be kept in mind as possible breeding goals to avoid potentially overlooking emerging priorities.

On the contrary, bull fertility and meat quality ranked quite low in terms of importance. A relatively new trait in local evaluation [14], bull fertility aims to improve reproduction potential and, secondly, growth ability (as mentioned by [38]). Scores assigned to meat quality possibly consider meat as sold by body weight rather than the quality of the meat itself. Such a payment system could explain the low economic value of this trait in both beef and dairy cattle production systems [17,39,40].

Herd clustering approaches

In our study, we applied ASW, WSS, and gap statistics as criteria for assessing clustering methods and determining cluster numbers. ASW or WSS were solely used for clustering herds of beef [6], dairy cattle [7], and sheep [41] for breeding purposes. Nevertheless, to the best of our knowledge, a combination of the various criteria for evaluating the performance of clustering approaches used in our study (ASW, WSS, and gap statistics) was applied in another study examining the general typology of small farms across Europe [3]. The authors considered ASW, connectivity, and the Dunn index to choose the most suitable algorithms for data clustering and gap statistics to define an optimal number of clusters. Contrary to previously mentioned papers, in our study, the ASW evaluation criteria showed separation efficiency similarity among clustering approaches and clearly did not prioritize one approach over another. The AHC method showed slight superiority with three clusters, which could fit our data. This presumption was confirmed when considering all evaluation criteria (ASW, WSS, and gap statistics, as indicated in the Results section). In our study, regarding the clustering method and number of clusters, a combination of various clustering criteria seems to be beneficial for finding the solution.

The separation similarity among the evaluated methods in Czech beef farms is supported by the fact that, when using the AHC and k-means as a function of three clusters, similar clusters were created. The only difference was farms switching between individual clusters (16/8/17 vs. 16/17/8 in cluster 1/2/3, respectively; see the plots in Supplementary Figure S1). Regarding the AHC and PAM approaches, the clustering results were identical for 76% of Czech beef farms. Such results also partly confirmed the general statement that these methods (mainly those of the partitioning group) work well for finding clusters in small- to medium-sized databases [1]. This was the case in our study (consisting of seven characteristics of 41 farms), as well as in that of [4], which evaluated about 300 animals with various Charolais gene ratios according to six variables. Likewise, 15 determinants were applied by the authors of [41] to cluster 25 sheep farms, and the AHC method was found to be the most suitable approach. Nonetheless, this approach seems to be suitable for evaluating larger datasets as well. The k-means approach was applied for clustering nearly 73 thousand beef calves based on their weaning weight and pedigree data [6]. Similarly, 22 production and economic variables in most small European territorial units were analyzed by the authors of [3], who found that the PAM approach is the most suitable solution for such data. It is worth noting that this method outperformed the centroid-based CLARA approach specified for large datasets. Generally speaking, centroid-based partitioning and hierarchical methods have successfully been used for clustering the various structures of farm data.

Regarding the overall and cluster-specific characteristics applied as determinants of Czech beef farms (breed, herd size, management and marketing strategy, farmer age), most of them were shared with other studies that clustered farms for breeding purposes by population level (e.g., [7,41]). More specific determinants (cow age, live weight, and body measurements) were considered to provide a phenotypic evaluation of beef herds [4]. Similarly, after herd size, some environmental factors (average temperature and rainfall) were also considered during clustering to investigate genotype–environment interactions [6]. Likewise, various production and economic determinants were taken into account to establish the basic typology and characteristics of suckler farm systems and evaluate their viability and economic perspectives [2]. In addition to classical characteristics, such as herd size and pasture area, they evaluated the degree of intensity, labor efficiency, housing system, and infrastructure level. In this context, some specific determinants, such as breeding strategy (pure/crossbreeding or mixed) and selection decision (based on BVs, performance, or both), that were incorporated into our survey aimed to define the overall Czech beef breeding system, specifically to further breeding goals and enhance indexes (indices).

Cluster characteristics, preferences, and breeding strategies

Based on the overall characteristics and preferences of farmers participating in the survey (both discussed above), they proved to be representative of Czech production conditions, supporting some of the top traits currently included in genetic evaluation and identifying some novel traits. Moreover, clustering could allow us to gain a more precise understanding of beef farms and breeding objectives from a breeding and selection point of view. As previously mentioned, production (breeding) system characteristics significantly influenced breeding goals and farm economics; secondly, farmer requirements regarding breeding animals may differ significantly according to their breeding objective [13].

The clustering provided in our study indicated three breeding systems in the Czech beef population, organized in terms of herd size, management, breeding strategy, breed structure, and farmer age. The same was true for the respective cluster preferences of breeding goal traits. BV and performance were the joint primary information sources in all three breeding systems. Cow temperament and calf viability, along with maternal fertility and longevity, were found to be among the most important mandatory cluster-specific traits. Although these are relatively novel traits in local genetic evaluation, they fully correspond to breeders’ overall interest in achieving sustainability and efficiency. Calf production per cow life, as a result of maternal fertility, calf viability, maternal temperament, and cow longevity, most likely plays a leading role in this case. Similarly, cow productivity was found to be one of the core economic parameters of Greek beef farms [2]. The authors further recognized that this was also connected to the level of veterinary and zootechnical support on pastures. In accordance with this statement, animal health was found to be a further high-scoring trait in all Czech beef clusters, ranging from 4.0 in clusters 2 and 3 to 5.0 in cluster 1 (as shown in Figure 5). Such a score difference between clusters was reported as large in terms of Cohen’s d value (Table S3) and indicated the practical trait importance for cluster 1. Health was not considered a mandatory trait in our survey, as it has not yet been incorporated into the ongoing genetic evaluation. Nevertheless, it was suggested as a new breeding goal directly by survey participants. The way it could contribute to further breeding was briefly discussed above (in the context of overall trait preferences); health should probably be validated when enhancing beef breeding goals for individual breeding systems. Nevertheless, the position of animal health as an important breeding objective was generally confirmed among livestock species [7,41,42].

In terms of breeding strategy, pure-breeding dominated in the farms of cluster 1; nonetheless, a combination of pure- and crossbreeding strategies was typical for the rest of the farm clusters. This parameter would probably be the first distinctive feature to define particular breeding systems and goals. Pure-breeding farms have specialized in producing breeding animals and, in part, other beef categories (such as weaned calves and/or fatteners (as by-products)). This breeding system seems to be fully compatible with two selection strategies focused on production sires as foundation bulls (sires of dams) and sires for beef herds, both formerly defined for Czech beef conditions [13]. In this study, breeding goals were further specified to gain heifers for replacement (with good maternal traits) and weaned/slaughter calves with excellent growth ability. In accordance with this intention, calving performance and growth were found to be among the top-scoring traits (3.94, Figure 5) in cluster 1 in our study. Across all three clusters, farmers paid higher attention to production traits (13% vs. 11% in cluster 2) and only placed growth among the top four breeding goal traits. Likewise, health received the highest score (5.0) when considering all trait preferences across clusters. Exterior, legs, body capacity, and production type were further high-scoring traits (3.75 to 3.88). The second characteristic of cluster 1 was a predominance of smaller herds, running conventionally and managed by younger farmers. The farmers’ age category and a lower herd size might partially align with conventional management and pure-breeding strategies, as these are appropriate in relation to their practical experiences and farm scales. Nevertheless, in some cases, the pure-breeding strategy would be based more on personal conviction than the farmer’s age.

Farms in clusters 2 and 3 combined pure- and crossbreeding strategies (100% and 88%, respectively). Incomes from breeding animals were characteristic of one-third of all farms (38% and 24% in clusters 2 and 3, respectively, as shown in Figure 4). The ratio of respondents focused on other beef categories was two to three times higher in comparison to purebred farms (e.g., 25% vs. 6% on weaned calves in cluster 2 vs. cluster 1). Therefore, overall production could be defined as partially (cluster 2) and fully (cluster 3) diversified, focusing on selling both breeding animals and other beef categories while using pure- and crossbreeding strategies. In view of the above findings, these farm clusters were also specific in terms of management strategy. Higher production differentiation was likely possible due to herd size (large and medium in clusters 2 and 3, respectively), whereby a portion of cows could be included in a crossbreeding strategy to produce other beef categories. One common characteristic was a predominance of ecological management (in contrast to the conventional farms prevailing in cluster 1), which probably carried certain limitations (e.g., in terms of land management and animal treatment). Such diversified production and specific production conditions are in agreement with the findings of study [2], where farmers genetically improved the production parameters of animals and final product quality. Nonetheless, this was all in accordance with the production conditions.

In terms of breed structure, two main groups were indicated. Cluster 2 represented farms that specified four of the most frequent breeds, whereas farms in cluster 3 also covered other breeds (Belgian Blue, Salers, and Galloway, making seven in total). In this context, breeds may differ according to the purpose they will be used for [13]. The same was true for scores assigned to potential breeding goals in clusters 2 and 3 in our study. After the top traits mentioned above, calving performance, growth, polledness, and udder score were among the most important traits in cluster 2. On the contrary, exterior traits (polledness, body capacity, productive type, muscularity, and legs) were among other most preferred goals in cluster 3. Their scores assigned by farmers in cluster 3 were noticeably or substantially different from scores in cluster 2 (see Table S3). Partially diversified farms in cluster 2 (38% purely produced breeding animals) paid higher attention to the traits currently defined among the top breeding goals, which are probably most appreciated when selling breeding animals. These criteria are therefore similar to those applied in cluster 1, in that they are entirely oriented to pure-breeding, in accordance with the above-mentioned selection strategy for producing sires for beef herds [13]. On the contrary, fully diversified farms in cluster 3 (24% having breeding animals as the only product and 12% represented by farms solely oriented to crossbreeding) were intensely focused on phenotype and animal performance. Pure-breeding is probably applied on these farms to produce breeding animals (especially cows) to replace their own herds. Therefore, farms in cluster 2 combined current top breeding traits and exterior traits; this is in contrast with farms in cluster 3, which altogether preferred exterior performance. The dominant position of exterior traits in cluster 3 was supported by the score assigned to the production (breeding) type (4.12), which was significantly higher than in cluster 2 (3.25). Production type has a high genetic correlation with overall animal muscularity and therefore could be used as a representative for exterior traits [13]. The same was true for the trait preferences when score for production type was moderately (0.56 on average) and significantly correlated to all exterior traits (Table S2). Regarding the main breeding goal categories, farmers in cluster 2 paid slightly higher attention to functional traits (39% vs. 35% in cluster 3), and farmers in cluster 3 preferred exterior (53% vs. 49% in cluster 1).

In the context of the main production, functional, and exterior trait categories, a slight preference for the latter was indicated by both, as indicated by their relatively high scores (all over 3.1 score; legs in cluster 2) and the highest number of traits (seven in total) involved. On the contrary, the production category covered only two traits (growth and meat quality), and for the second trait, the score was typically very low (1.7 to 2.7 in clusters 2 and 1, respectively). The functional category showed a moderate emphasis, covering five traits in total and four with relatively high scores in all clusters (3.7 for calving performance in cluster 3 to 5.0 for health in cluster 1). These specifics determined the overall superiority of the exterior category (51% on average). On the contrary, in case the trait numbers among these categories were more balanced and health traits were separated from the functional category (e.g., 3:4:3:2 traits in sheep breeding systems [41]), the individual trait and overall category importance could possibly be understood more clearly (e.g., 22:32:29:17 ratio [41]). For Czech beef breeding purposes, future studies could reassess traits in the functional (specification of those related to health) and exterior (omitting the body frame due to low preferences, defining the production type as representative (as mentioned above)) categories.

Our survey was predominantly oriented toward breeds in the beef breeding system. Nevertheless, based on recent communication with local breeders, they are also interested in crossbreeding beef bulls with dairy cows (i.e., beef on dairy system). This system has already been mentioned in an earlier Czech study [13]; however, specific breeding objectives and selection candidates have not been defined, and no practical applications for mating plans have been provided. Nevertheless, the number of Czech Holstein cows included in this breeding strategy increased three times in the last decade (up to nearly three thousand matings in 2023, based on our own calculations from the database provided by the CBCA). To identify beef bulls suitable for such a strategy, breeding objectives (e.g., easy calving, short gestation) should be developed and appropriately validated [43]. Moreover, some financial benefits of the system indicated by [43] would probably be linked to diversified production and enhanced sources of revenues, as found in our study (Figure 4) and others already cited [31,32].

Our further investigation will reflect the cluster-specific patterns in breeding systems and breeding objective trait preferences to inform tailored breeding programs for Czech beef cattle conditions. The current breeding program, which is universal and simple, will be modified to consider farm specifics in marketing and breeding strategies (pure/cross breeding, specialization vs. part/fully diversification, beef on dairy), farmed breed(s) with new functional traits among the top breeding objectives, and selecting mostly on performance (growth, calving performance) vs. exterior traits. The trait categories in breeding objectives will be more balanced (e.g., separate health from functional category). Similarly, the economic weights of beef breeding goal traits calculated previously [17] will be updated to consider cluster-specific characteristics and preferences. Farm characteristics will ensure the appropriate setting of production systems, and preferences for the scope of traits will be economically evaluated in the bio-economic model. The current farmers’ interests in terms of beef on dairy strategy will focus on the genetic and economic evaluation of breeding objective traits for such a breeding system under local conditions (as part of the ongoing project). The production (genetic) and economic benefits of the suggested breeding strategies and considered traits will be evaluated. All features will be discussed with the local beef cattle association and breeders (farmers) to apply them into selection in a practically appropriate way.

## 5. Conclusions

Participating beef cattle farms proved to be representative of Czech conditions in terms of applied management, marketing, breeding strategies, and farmed breeds. The herd size structure was balanced, covering both smaller and larger producing farms. The average farmer age corresponded to the general agriculture employee structure, partially indicating a generational change. For animal breeding and selection, farmers considered both breeding values and animal performance, which aids in building a foundation for further breeding program enhancements and goal definition. Overall farmer preferences correspond to the top traits considered in current local genetic evaluation (calving performance, growth, and exterior), with relatively high emphasis on novel traits (cow temperament and calf viability, maternal fertility and longevity) and the suggestion of optional traits (polledness and animal health) for potential breeding objectives representing welfare, sustainable, and security attributes.

Applying various clustering approaches revealed similarity in terms of farm separation efficiency, showing a slight preference for agglomerative hierarchical clustering as the most suitable approach for fitting the data. Three clusters were suggested, specified according to herd size, management, marketing, breeding strategy, breed structure, and farmer age. The same was true for the respective cluster preferences of breeding goal traits. Farms in cluster 1 specialized in pure-breeding and the production of breeding animals, whereas farms in other clusters combined pure- and crossbreeding strategies with partially (cluster 2) and fully (cluster 3) diversified production across all beef categories. Among the top-scoring traits were calving performance and growth (clusters 1 and 2) and exterior (cluster 3). Most of the top breeding goal traits were relatively new in the local evaluation. Nevertheless, they could have an essential place in local breeding. Understanding and consideration of local beef breeding system characteristics and farmers’ breeding preferences will be applied in further enhancing breeding programs. These findings will inform breeding programs to enhance genetic progress and sustainability in Czech beef production.

In the context of the methodological approach presented in our study, it could be widely applied for further populations and production conditions; a combination of various clustering methods and evaluation criteria may be helpful to find the most appropriate solution for the analyzed population dataset. A valuable overall and cluster-specific pattern could be identified from the online questionnaire dataset source and size. As the most relevant determinants, management and marketing strategies, herd size, and farmed breed(s) could be considered. Regarding the clustering approach applied in our study and across the literature cited, centroid-based partitioning and hierarchical methods enable the successful clustering of various structures of farm datasets.

## Figures and Tables

**Table 1:** Structure of beef cattle farm characteristics and overall survey responses.

Farm Characteristics (Variables)	Category	Responses (%)
(1) Management	Conventional	44
	Ecological	49
	In transition	2
	Mixed (conventional + ecology)	5
(2) Marketing strategy (predominant type of sale)	Breeding animals	34
	Weaned calves	17
	Fattening	15
	Breeding animals + weaning or fattening	29
	Weaning + fattening	5
(3) Breeding strategy	Pure-breeding	39
	Crossbreeding	5
	Both strategies	56
(4) Breed in the herd (in total/the first, i.e., dominant)	Aberdeen angus	25/25
	Limousine	25/19
	Charolais	17/15
	Beef Simmental	13/6
	Belgian Blue	4/4
	Salers	4/2
	Hereford	4/4
	Piemontese	2/2
	Pinzgau	2/0
	Aubrac	2/0
	Galloway	2/2
(5) Herd size group (number of cows/herd)	≤29	22
	30 to 59	15
	60 to 89	17
	90 to 119	10
	120 to 149	5
	150 to 179	10
	180 to 209	2
	210 to 239	7
	≥240	12
(6) Farmer age group (in years)	≤39	34
	40 to 49	41
	50 to 59	15
	60 to 69	7
	≥70	2
(7) Breeding and selection decision	Breeding values (BVs)	9
	Achieved performance	18
	Both BVs and performance	73
	Other ^1^	0

^1^ The farmer added their own answer.

**Table 2:** Mandatory and optional traits evaluated by beef cattle farmers.

Trait Group ^1^ (Abbreviation)/Category ^2^		Traits Included
Calving performance (CP)	F	calving difficulty, birth weight
Growth (GR)	P	live weight at the age of 120, 210, and 365 days, daily bull gain in the test
Body frame (BoF)	E	height at sacrum, body length, live weight
Capacity (CA)	E	front chest width, chest depth, pelvis length
Muscularity (MU)	E	shoulder, back (top line), rump
Production (breeding) type (PT)	E	overall body shaping, body conformation balance, sex expression
Legs (LE)	E	front leg positions, fore pastern length and angle, hind leg positions, hind leg angle, hind pastern length and angle
Maternal fertility and longevity (MF_Lon)	F	age at first calving, first calving interval, longevity
Udder scoring (UD)	E	udder shape, teat length, teat width and shape, depth (size), any udder defects
Temperament of cow and viability of calf (Tem_Vi)	F	temperament in relation to the breeder and to the calf, viability of the calves
Bull fertility (BuF)	F	scrotal circumference
Meat quality (MQ)	P	ultrasonic measurements
Optional traits ^3^ Polledness (Poll) Animal health (Health)	E F	optionally added by farmers

^1^ A detailed description of the traits included has been published in previous studies [14,15,24,25,26] and on the Czech Beef Cattle Association (CBCA) web pages (in Czech; https://www.cschms.cz/index.php?page=sle_info, accessed on 21 July 2025) in accordance with the International Committee for Animal Recording (ICAR) Guidelines [27]. ^2^ Trait category: P—production, F—functional, E—exterior. ^3^ The most frequent traits (polledness and animal health, added by ten and eight farmers, respectively) were considered in our evaluation. Traits with low frequency (gestation length, feed conversion, and body condition added by 4 farmers in total) were omitted from further evaluation.

## Data Availability

Data are available upon request to the corresponding author.

## Supplementary Materials

The following supporting information can be downloaded at https://www.mdpi.com/article/10.3390/ani15152175/s1. Table S1: Correlation coefficients (upper) and corresponding *p*-values (lower) among the herd characteristics and farmers’ preferences for breeding objective traits (variables); Table S2: Correlation coefficients (upper) and corresponding *p*-values (lower) among the farmers’ preferences for breeding objective traits (variables). Table S3: Pairwise analyses of the trait score differences between clusters in terms of effect sizes measured by the Cohen´s d values. Figure S1: Average silhouette width within herds and clusters as a function of three clusters using the k-means, partition around medoids (PAM), and agglomerative hierarchical clustering (AHC) approaches, respectively.
