# Supplementary material for: Beef Breeding Systems and Preferences for Breeding Objective Traits
Source: Animals (Basel). 2025 Jul 23;15(15):2175. doi: 10.3390/ani15152175 (PMC12345501; doi:10.3390/ani15152175)
Supplement: Supplementary file 1 [file animals-15-02175-s001.zip › animals-3728478-supplementary.pdf]

animals

MDPI

Supplement

Beef Breeding Systems and Preferences for Breeding Objective Traits

Zuzana Krupová [1,*], Emil Krupa [1], Michaela Brzáková [1], Zdeňka Veselá [1] and Kamil Malát [2]

Table S1. Correlation coefficients (upper) and corresponding p-values [1] (lower) among the herd characteristics and farmers' preferences for breeding objective traits (variables) [2]

Variable [2]	managm	market	pureb_n	cross_n	pureb_1	cross_1	herd_s	age_gr	decis
managm	1.000	-0.025	**0.424**	**0.356**	-0.017	0.186	0.248	0.044	0.032
		0.8774	**0.0057**	**0.0223**	0.9172	0.2451	0.1184	0.7824	0.8430
market	-0.025	1.000	0.191	0.051	-0.116	0.111	**0.350**	0.103	-0.200
	0.8774		0.2320	0.7497	0.4692	0.4911	**0.0249**	0.5234	0.2101
pureb_n	**0.424**	0.191	1.000	0.298	-0.122	0.165	0.257	0.083	0.042
	**0.0057**	0.2320		0.0587	0.4457	0.3030	0.1043	0.6075	0.7943
cross_n	**0.356**	0.051	0.298	1.000	-0.241	**0.507**	**0.418**	0.174	-0.223
	**0.0223**	0.7497	0.0587		0.1298	**0.0007**	**0.0065**	0.2752	0.1618
pureb_1	-0.017	-0.116	-0.122	-0.241	1.000	-0.214	-0.236	-0.272	0.272
	0.9172	0.4692	0.4457	0.1298		0.1798	0.1367	0.0858	0.0858
cross_1	0.186	0.111	0.165	**0.507**	-0.214	1.000	0.141	0.243	-0.116
	0.2451	0.4911	0.3030	**0.0007**	0.1798		0.3809	0.1259	0.4706
herd_s	0.248	**0.350**	0.257	**0.418**	-0.236	0.141	1.000	0.007	-0.086
	0.1184	**0.0249**	0.1043	**0.0065**	0.1367	0.3809		0.9633	0.5940
age_gr	0.044	0.103	0.083	0.174	-0.272	0.243	0.007	1.000	-0.193
	0.7824	0.5234	0.6075	0.2752	0.0858	0.1259	0.9633		0.2274
decis	0.032	-0.200	0.042	-0.223	0.272	-0.116	-0.086	-0.193	1.000
	0.8430	0.2101	0.7943	0.1618	0.0858	0.4706	0.5940	0.2274	
CP	0.277	0.174	0.081	0.161	-0.253	-0.142	-0.050	-0.085	-0.051
	0.0796	0.2763	0.6126	0.3136	0.1102	0.3772	0.7546	0.5975	0.7502
GR	-0.017	-0.104	0.102	0.063	0.173	0.077	-0.130	-0.069	0.167
	0.9153	0.5180	0.5262	0.6978	0.2808	0.6302	0.4181	0.6686	0.2959
BoF	0.025	-0.230	-0.193	-0.043	0.115	0.198	**-0.311**	-0.042	0.104
	0.8760	0.1489	0.2267	0.7915	0.4751	0.2145	**0.0476**	0.7936	0.5192
CA	0.014	-0.287	-0.181	-0.065	-0.013	0.102	**-0.351**	0.029	-0.003
	0.9308	0.0689	0.2563	0.6876	0.9334	0.5257	**0.0243**	0.8565	0.9854
MU	0.006	-0.180	-0.180	0.078	0.061	0.049	-0.236	0.305	-0.036
	0.9704	0.2606	0.2589	0.6295	0.7050	0.7614	0.1376	0.0528	0.8240
PT	0.015	-0.209	**-0.396**	-0.171	-0.105	0.102	-0.240	0.150	0.180
	0.9265	0.1905	**0.0104**	0.2839	0.5154	0.5258	0.1311	0.3487	0.2601
LE	-0.092	-0.115	-0.196	-0.174	-0.213	0.098	0.013	-0.081	-0.057
	0.5665	0.4734	0.2202	0.2754	0.1802	0.5433	0.9371	0.6153	0.7219
MF_Lon	0.201	0.168	0.202	0.079	0.159	0.040	-0.107	-0.046	0.092
	0.2066	0.2949	0.2050	0.6213	0.3204	0.8046	0.5065	0.7756	0.5663
UD	0.211	-0.065	-0.043	0.234	**-0.391**	-0.091	0.072	0.107	-0.020
	0.1857	0.6854	0.7876	0.1415	**0.0115**	0.5734	0.6549	0.5047	0.9002
Tem_Vi	0.102	-0.192	-0.006	0.038	-0.037	-0.204	-0.269	-0.177	-0.167
	0.5244	0.2303	0.9687	0.8128	0.8171	0.2014	0.0895	0.2684	0.2967

**Bu_F**	0.284	0.203	0.266	0.287	**-0.423**	0.038	0.182	0.146	-0.039
	0.0723	0.2020	0.0928	0.0689	**0.0058**	0.8144	0.2542	0.3613	0.8093
**MQ**	0.108	0.269	-0.114	-0.130	-0.037	-0.244	0.028	-0.072	-0.055
	0.5014	0.0888	0.4769	0.4184	0.8168	0.1238	0.8626	0.6551	0.7350
**Pool**	0.031	-0.211	-0.097	0.059	-0.020	0.004	-0.048	-0.096	-0.120
	0.8463	0.1858	0.5452	0.7136	0.9027	0.9803	0.7666	0.5487	0.4537
**Heal**	0.187	-0.029	-0.075	-0.067	-0.076	-0.030	-0.065	-0.090	0.078
	0.2411	0.8590	0.6417	0.6784	0.6383	0.8501	0.6882	0.5771	0.6279

[1] Pearson correlation coefficients and p-values calculated for variables of 41 farms by the "rcorr" function in the "Hmisc" R package [23]; significant correlations with P <0.05 printed in bold.

[2] "managm" – management (e.g. conventional, ecological), "market" – marketing strategy (e.g. selling of breeding animals, weaned calves, fatteners), "pureb" and "cross" – breeding strategy (pure and cross breeding, or both) both labelled with "n" – number of breeds used in total and "1" breed defined by farmer as the first (i.e. considered as a dominant breed for the given herd), "herd_s" – herd size group (number of cows), "age_gr" – age group of a farmer (social aspect) and "decis" – general decision when selecting animals (i.e. based on performance, BV, or both). CP—calving performance, GR—growth, BoF—body frame, CA—capacity, MU—muscularity, PT—production (breeding) type, LE—legs, MF_Lon—maternal fertility and longevity, UD—udder scoring, TEM_Vi—cow temperament and calf viability, BuF—bull fertility, MQ—meat quality, Poll—polledness and Health—animal health.

Table S2. Correlation coefficients (upper) and corresponding p-values [1] (lower) among the farmers' preferences for breeding objective traits (variables) [2]

**Variable** [2]	**CP**	**GR**	**BoF**	**CA**	**MU**	**PT**	**LE**	**MF_Lon**	**UD**	**Tem_Vi**	**Bu_F**	**MQ**	**Pool**	**Heal**
**CP**	1.000	0.061	0.179	0.170	0.124	0.080	0.155	0.115	**0.478**	**0.596**	**0.359**	0.308	0.218	0.300
		0.7062	0.2616	0.2891	0.4411	0.6192	0.3335	0.4747	**0.0016**	**0.0000**	**0.0212**	0.0504	0.1708	0.0565
**GR**	0.061	1.000	**0.384**	0.308	0.292	0.131	0.006	0.021	-0.194	0.061	-0.190	-0.131	0.034	0.272
	0.7062		**0.0131**	0.0503	0.0639	0.4139	0.9693	0.8979	0.2250	0.7039	0.2341	0.4150	0.8336	0.0857
**BoF**	0.179	**0.384**	1.000	**0.744**	**0.576**	**0.535**	**0.404**	-0.061	-0.035	0.179	**-0.313**	**-0.329**	**0.486**	0.264
	0.2616	**0.0131**		**0.0000**	**0.0001**	**0.0003**	**0.0087**	0.7050	0.8275	0.2625	**0.0467**	**0.0360**	**0.0013**	0.0952
**CA**	0.170	0.308	**0.744**	1.000	**0.723**	**0.595**	**0.496**	0.003	0.063	**0.348**	-0.132	-0.166	**0.356**	**0.376**
	0.2891	0.0503	**0.0000**		**0.0000**	**0.0000**	**0.0010**	0.9851	0.6973	**0.0259**	0.4108	0.3011	**0.0222**	**0.0154**
**MU**	0.124	0.292	**0.576**	**0.723**	1.000	**0.555**	**0.314**	-0.128	0.025	0.156	-0.074	-0.071	0.139	0.237
	0.4411	0.0639	**0.0001**	**0.0000**		**0.0002**	**0.0454**	0.4263	0.8788	0.3310	0.6440	0.6601	0.3848	0.1352
**PT**	0.080	0.131	**0.535**	**0.595**	**0.555**	1.000	**0.545**	0.075	0.037	0.117	-0.005	-0.060	0.093	0.216
	0.6192	0.4139	**0.0003**	**0.0000**	**0.0002**		**0.0002**	0.6420	0.8199	0.4666	0.9735	0.7076	0.5643	0.1760
**LE**	0.155	0.006	**0.404**	**0.496**	**0.314**	**0.545**	1.000	0.129	0.093	0.273	0.137	-0.205	0.285	0.105
	0.3335	0.9693	**0.0087**	**0.0010**	**0.0454**	**0.0002**		0.4219	0.5625	0.0842	0.3931	0.1976	0.0710	0.5124
**MF_Lon**	0.115	0.021	-0.061	0.003	-0.128	0.075	0.129	1.000	0.085	0.285	0.231	-0.070	-0.067	-0.174
	0.4747	0.8979	0.7050	0.9851	0.4263	0.6420	0.4219		0.5963	0.0712	0.1460	0.6618	0.6756	0.2769
**UD**	**0.478**	-0.194	-0.035	0.063	0.025	0.037	0.093	0.085	1.000	**0.379**	**0.371**	-0.004	-0.050	0.143
	**0.0016**	0.2250	0.8275	0.6973	0.8788	0.8199	0.5625	0.5963		**0.0145**	**0.0168**	0.9785	0.7582	0.3729
**Tem_Vi**	**0.596**	0.061	0.179	**0.348**	0.156	0.117	0.273	0.285	**0.379**	1.000	0.263	-0.097	0.150	0.155
	**0.0000**	0.7039	0.2625	**0.0259**	0.3310	0.4666	0.0842	0.0712	**0.0145**		0.0965	0.5473	0.3484	0.3341
**Bu_F**	**0.359**	-0.190	**-0.313**	-0.132	-0.074	-0.005	0.137	0.231	**0.371**	0.263	1.000	0.278	-0.296	-0.057
	**0.0212**	0.2341	**0.0467**	0.4108	0.6440	0.9735	0.3931	0.1460	**0.0168**	0.0965		0.0783	0.0603	0.7231
**MQ**	0.308	-0.131	**-0.329**	-0.166	-0.071	-0.060	-0.205	-0.070	-0.004	-0.097	0.278	1.000	**-0.322**	0.064
	0.0504	0.4150	**0.0360**	0.3011	0.6601	0.7076	0.1976	0.6618	0.9785	0.5473	0.0783		**0.0403**	0.6890
**Pool**	0.218	0.034	**0.486**	**0.356**	0.139	0.093	0.285	-0.067	-0.050	0.150	-0.296	**-0.322**	1.000	0.243
	0.1708	0.8336	**0.0013**	**0.0222**	0.3848	0.5643	0.0710	0.6756	0.7582	0.3484	0.0603	**0.0403**		0.1260
**Heal**	0.300	0.272	0.264	**0.376**	0.237	0.216	0.105	-0.174	0.143	0.155	-0.057	0.064	0.243	1.000
	0.0565	0.0857	0.0952	**0.0154**	0.1352	0.1760	0.5124	0.2769	0.3729	0.3341	0.7231	0.6890	0.1260	

[1] Pearson correlation coefficients and p-values calculated for variables of 41 farms by the "rcorr" function in the "Hmisc" R package [23]; significant correlations with P <0.05 printed in bold.
[2] CP—calving performance, GR—growth, BoF—body frame, CA—capacity, MU—muscularity, PT—production (breeding) type, LE—legs, MF_Lon—maternal fertility and longevity, UD—udder scoring, TEM_Vi—cow temperament and calf viability, BuF—bull fertility, MQ—meat quality, Poll—polledness and Health—animal health.

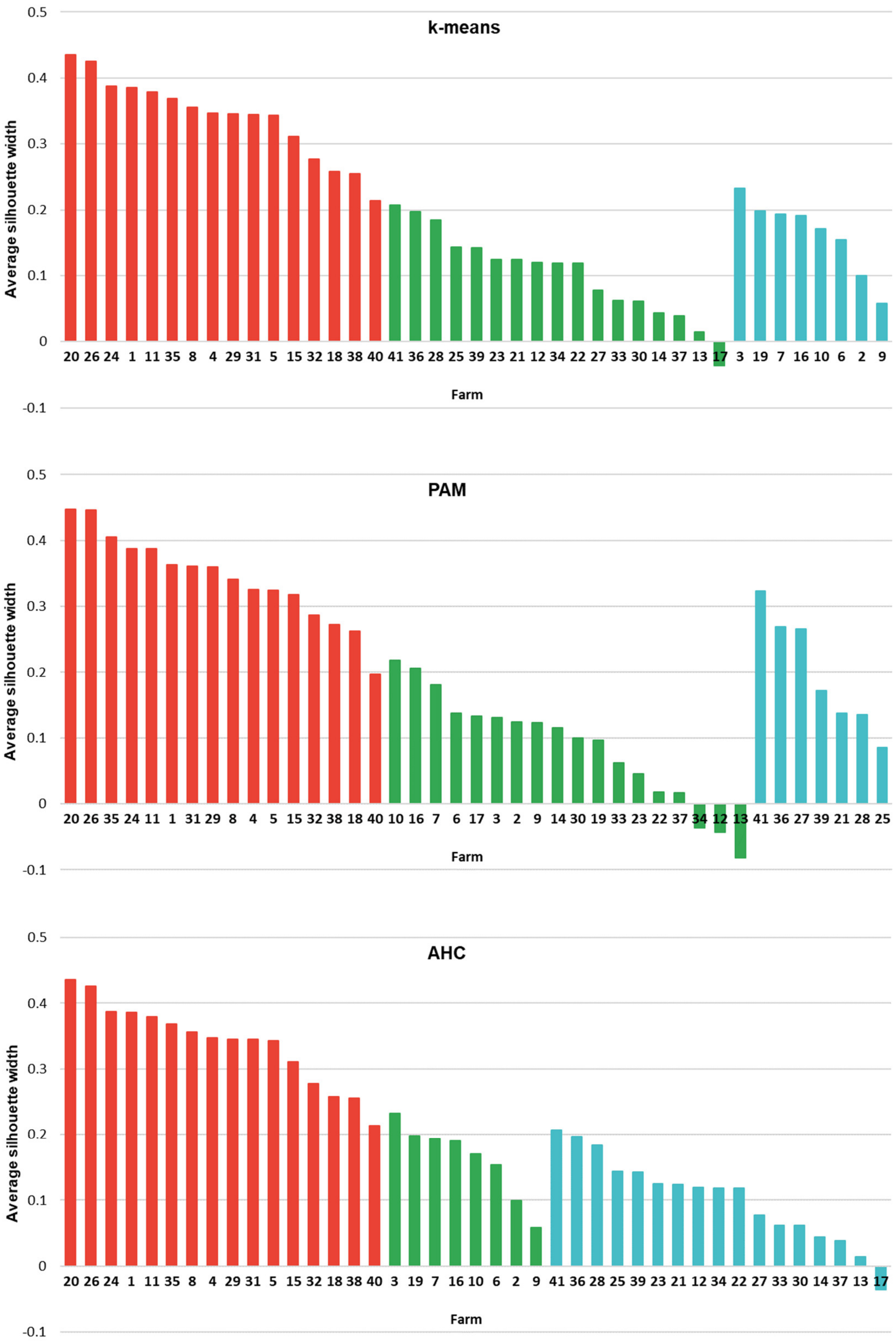

Figure S1. Average silhouette width within herds and clusters as a function of three clusters using the k-means, partition around medoids (PAM), and agglomerative hierarchical clustering (AHC) approach, respectively.

 https://doi.org/10.3390/ani15152175

Table S3. Pairwise analyses of the trait score differences between clusters in terms of effect sizes measured by the Cohen´s d values [1].

Trait [2]	Cluster 1 vs. 2			Cluster 1 vs. 3			Cluster 2 vs. 3		
	Cohen's d	95% CI		Cohen's d	95% CI		Cohen's d	95% CI	
		lower	upper		lower	upper		lower	upper
**CP**	0.225	-0.676	1.126	0.175	-0.537	0.089	0.468	-0.429	1.366
**GR**	0.151	-0.748	1.050	0.042	-0.669	0.752	0.210	-0.679	1.099
**BoF**	-0.154	-0.898	0.898	-0.270	-0.990	0.437	-0.350	-1.242	0.543
**CA**	-0.217	-1.118	0.682	-0.470	-1.190	0.249	-0.799	-1.717	0.118
**MU**	-0.201	-1.002	0.695	-0.526	-1.249	0.196	-0.756	-1.670	0.158
**PT**	-0.474	-1.383	0.435	-0.347	-1.063	0.368	**-1.001**	-1.936	-0.067
**LE**	-0.566	-1.479	0.348	-0.204	-0.865	0.321	**-0.849**	-1.770	0.072
**MF_Lon**	0.482	-0.427	1.392	-0.208	-0.924	0.528	0.384	-0.509	1.278
**UD**	0.728	-0.196	1.651	-0.025	-0.735	0.685	0.644	-0.263	1.550
**Tem_Vi**	0.054	-0.898	0.828	0.090	-0.621	0.800	0.094	-0.793	0.982
**BuF**	0.620	-0.297	1.536	-0.267	-0.980	0.448	0.413	-0.482	1.308
**MQ**	0.670	-0.269	1.664	**1.438**	0.412	2.464	**1.578**	0.765	2.392
**Poll**	**-1.632**	-4.371	1.105	0.277	-2.137	2.692	**-0.949**	-2.774	0.876
**Health**	**0.929**	-0.247	2.106	-0.467	-1.601	0.665	-0.424	-1.508	0.660

[1] Cohen´s d value, lower and upper confidence intervals (CI) of the difference between mean score of traits in the respective clusters calcualted by the R Statistical Software version 4.4.3 [18]. Cohen´s d (absolute) value: 0.2 - small effect (subtle difference), 0.5 - medium effect (noticeable difference), 0.8 - large effect (substantial difference; printed in bold). For more detailed description of clusters, please see Figure 4 and 5, and Table S1a,b.

[2] CP—calving performance, GR—growth, BoF—body frame, CA—capacity, MU—muscularity, PT—production (breeding) type, LE—legs, MF_Lon—maternal fertility and longevity, UD—udder scoring, TEM_Vi—cow temperament and calf viability, BuF—bull fertility, MQ—meat quality, Poll—polledness and Health—animal health.
